# Causes and outcomes of revision surgery in subjects with pulsatile tinnitus

**DOI:** 10.3389/fneur.2023.1215636

**Published:** 2023-07-24

**Authors:** Ye Ji Shim, Hanju Lee, Sung-Min Park, Dohee Kim, Ja-Won Koo, Jae-Jin Song

**Affiliations:** ^1^Department of Otorhinolaryngology-Head and Neck Surgery, Healthcare System Gangnam Center, Seoul National University Hospital, Seoul, Republic of Korea; ^2^Department of Otorhinolaryngology-Head and Neck Surgery, Seoul National University Hospital, Seoul, Republic of Korea; ^3^Department of Otorhinolaryngology-Head and Neck Surgery, Seoul National University Bundang Hospital, Seongnam, Republic of Korea

**Keywords:** pulsatile tinnitus, tinnitus, revision surgery, sigmoid sinus, jugular bulb, arteriovenous fistula

## Abstract

**Introduction:**

Once the underlying pathology has been identified, pulsatile tinnitus (PT) can be treated successfully with surgical or interventional management. However, some patients experience residual or recurrent symptoms following initially successful surgical treatment, and require revision surgery or additional procedures. Here, we report a case series of patients who had undergone revision surgery or interventional treatment, and suggest possible ways of minimizing the need for revision.

**Methods:**

Between January 2014 and March 2023, a total of seven subjects underwent revision surgery or interventional treatment for persistent or recurrent PT after initial surgical treatment. Demographic data, reasons for revision, and changes in symptoms before and after revision were analyzed retrospectively. Temporal bone computed tomographic angiography images were reviewed to identify the causes and reasons for revision.

**Results:**

Of the seven subjects, six underwent sigmoid sinus (SS) resurfacing/reshaping due to ipsilateral diverticulum (Div) or dehiscence (Deh), and one underwent jugular bulb (JB) resurfacing due to a high-riding JB with bony Deh. Of the five subjects who underwent revision SS surgery due to recurrent SS-Div or SS-Deh, three showed marked resolution of PT, while the other two showed partial improvement of the symptoms. One subject who underwent revision JB resurfacing, and another who underwent additional transarterial embolization for a concurrent ipsilateral dural arteriovenous fistula, reported marked improvement of PT.

**Discussion:**

The possibility of recurrence should be taken into account when performing surgical intervention in patients with PT. The likelihood of recurrence can be minimized through a comprehensive evaluation to identify possible multiple etiologies, and through the use of durable materials and appropriate surgical methods.

## 1. Introduction

Pulsatile tinnitus (PT) is a type of tinnitus characterized by a rhythmic sound in the ear, often synchronized with the heartbeat (1). The three major mechanisms by which vascular abnormalities may cause PT are alterations in vascular hemodynamics (causing turbulent blood flow leading to sound transmission), vibration of a dehiscent vascular wall in the absence of turbulent flow, and abnormal sound perception of a normal internal stimulus, such as in third-window lesions in the inner ear (2, 3). Therefore, identification of the causative vascular pathology and etiology-specific management are crucial for optimal treatment of PT, as adequate management targeting structural anomalies can result in complete resolution of PT (4, 5).

Surgical treatment can be effective for alleviating PT caused by vascular abnormalities revealed by radiological evaluation. Resurfacing/reshaping surgeries have been reported to improve PT in 70–100% of patients with sigmoid sinus (SS) anomalies, and there have also been several reports of the effects of resurfacing the jugular bulb (JB) anomalies (3, 6–9). Despite surgical correction of structural anomalies, however, PT may remain in some cases. In addition, even in cases of initially successful surgery, PT may recur during postoperative follow-up. A review article reported a 3.5% incidence of recurrent symptoms following initially successful surgical treatment for SS anomalies, and Li et al. (10) documented seven cases of revision surgery (7, 10). In previous studies, multiple vascular abnormalities with partial resolution in the initial surgery, insufficient understanding of venous sinus stenosis, and insufficient understanding of the spatial and stereoscopic structure of SS diverticulum or dehiscence have been proposed as the causes of PT recurrence necessitating revision surgery (10).

It is crucial to gain an understanding of the potential risk factors and reasons for residual symptoms or recurrence, to allow the refinement of treatment strategies for patients with PT due to vascular abnormalities. Here, we discuss cases of PT resulting from vascular anomalies that required revision surgery, along with the underlying reasons for persistence or recurrence despite initial surgical success.

## 2. Materials and methods

### 2.1. Patients

**Figure 1:**
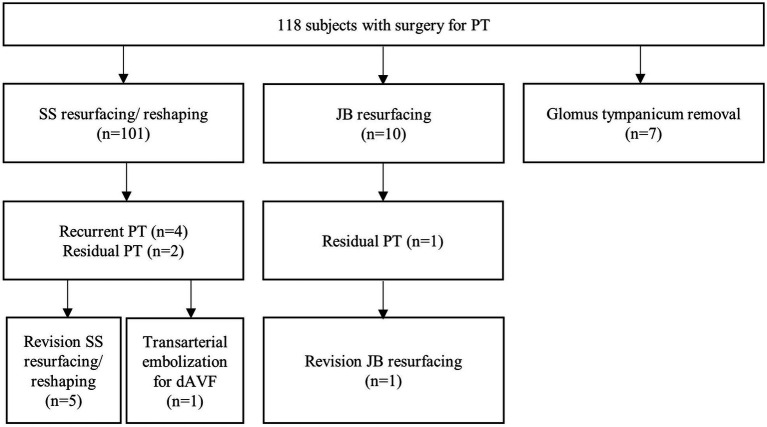
A schematic illustration of the subject classification who underwent revision surgery or interventional treatments for persistent or recurrent pulsatile tinnitus after the initial surgery. PT, pulsatile tinnitus; SS, sigmoid sinus; JB, jugular bulb; dAVF, dural arteriovenous fistula.

We retrospectively reviewed 118 subjects who underwent surgery for PT performed by a single surgeon at Seoul National University Bundang Hospital between January 2014 and March 2023. Out of 118 subjects, seven (5.9%) underwent revision surgery or interventional treatments for persistent or recurrent PT after the initial surgery (Figure 1). Of 101 subjects who underwent SS resurfacing or reshaping, 5 underwent revision SS surgery and one underwent an additional intervention for another vascular pathology. Among these six subjects, 1 underwent initial surgery at another hospital followed by revision surgery at Seoul National University Bundang Hospital. Of the 10 subjects who underwent JB resurfacing, 1 underwent revision surgery. None of the seven subjects diagnosed with glomus tympanicum required revision surgery or intervention. This study was approved by the Institutional Review Board (IRB) of the Clinical Research Institute at Seoul National University Bundang Hospital (IRB No. B-2305-830-101).

### 2.2. Radiological analysis

All subjects underwent temporal bone computed tomographic angiography (TB-CTA) (Philips Medical Systems, Eindhoven, The Netherlands). The scanning parameters used were as follows: 2 × 0.625-mm detector configuration; 0.7-mm slice thickness; 160 × 160 field of view; 220 mA; 120 kVp; and a 512 × 512 matrix. SS diverticulum (SS-Div) was defined as a partial protrusion of the SS into the mastoid air cells and thinning or loss of the calvarial cortex over the SS (3, 11). SS wall dehiscence (SS-Deh) was defined as direct contact between the SS and mastoid air cells due to thinning of the mastoid cortex and loss of hyperdense bony septa covering the SS, confirmed by at least two consecutive 0.7-mm cuts on axial TB-CTA images (3, 12). One subject underwent magnetic resonance angiography (MRA) after initial surgery and a concurrent dural arteriovenous fistula (dAVF) was identified. MRI was performed using a 3 T instrument (Achieva and Ingenia; Philips Healthcare, Best, the Netherlands) with a 32-channel SENSE Head Coil (Philips Healthcare). The following sequences were obtained: axial T2WI of the whole brain (FOV, 185 × 230 mm^2^; acquisition matrix size, 420 × 375; slice thickness, 5 mm; slice gap, 1 mm; NEX, 1; TR, 3000 ms; TE, 80 ms; flip angle, 90°), axial T1WI of the IAC and CPA (FOV, 180 × 180 mm^2^; acquisition matrix size, 272 × 217; slice thickness, 3 mm; slice gap, 0 mm; NEX, 1; TR, 500 ms; TE, 10 msec; flip angle, 50°), and 3D gadolinium-enhancing T1WI of the IAC and CPA (FOV, 200 × 200 mm^2^; acquisition matrix size, 256 × 256; slice thickness, 1 mm; slice gap, 1 mm; NEX, 1; TR, 9.5 ms; TE, 3.3 ms; flip angle, 8°). A high jugular bulb with bony dehiscence (HJBD) was defined as the JB extending to the level of the internal auditory canal without hyperdense bony septa between the tympanic cavity and JB (13, 14).

### 2.3. Surgical intervention

The detailed surgical procedures were described in our previous reports (3, 4, 6, 12, 15). Briefly, for SS resurfacing, the SS and its Div were skeletonized during cortical mastoidectomy. For JB resurfacing, the dehiscent portion was widely exposed via the transtympanic approach. Bony defects of the SS or JB were reconstructed by extraluminal application of various materials, including tissue sealant (TachoSil; Nycomed, Linz, Austria), temporalis fascia, and Mimix hydroxyapatite bone cement (W. Lorenz Surgical, Jacksonville, FL). For SS reshaping, an autologous cortical bone chip was inserted between the thinned bony shell of the SS and SS vessel wall to decompress the SS. The extent of the compression is always limited to less than 20% of the preoperative diameter of the SS not to cause postoperative increased intracranial pressure and related complications (16). Then, the dehiscent or thinned bony wall was reconstructed using a piece of temporalis fascia and Mimix bone cement, as described previously (15).

### 2.4. Evaluation of subjective symptoms

Subjective symptoms were evaluated before and after initial and revision surgery or interventional treatment. Subjective severity of tinnitus was assessed using the Tinnitus Handicap Inventory (THI) ranging from 0 to 100, and visual analog scale (VAS)-scored tinnitus loudness and tinnitus-related distress ranging from 0 to 10 (17). Despite the limited availability of validation studies on THI and other scales concerning PT patients, the utilization of THI and VAS scales for assessing treatment outcomes in this study aligns with established practices observed in previous studies (12, 15).

## 3. Results

### 3.1. Preoperative findings before the initial surgical intervention

The demographic and clinical characteristics of the seven subjects who underwent revision surgery or interventional treatment are summarized in Table 1. The median age was 41 years (39.5–46 years), and five of the seven subjects were female. All but one of the seven subjects presented with right-sided PT. The median preoperative THI score was 58 (37.5–83). The median tinnitus loudness and tinnitus-related distress scored on a VAS were 8 (7.5–9) and 9.5 (7.5–10), respectively. On preoperative TB-CTA, Subject 1 presented with SS-Div, Subjects 5 and 7 with SS-Deh, and Subjects 2, 3, and 6 showed combined SS-Div/SS-Deh on the affected side. Subjects 1, 2, and 6 showed diverticula extending into the mastoid cortex, while Subject 3 showed a Div protruding through mastoid air cells. Subject 6 presented with a large Div that protruded through the mastoid air cells and mastoid cortex and reached the subcutaneous tissue. Subject 7 had a large bony Deh at the transverse-sigmoid junction area. Subject 4 showed HJBD with dehiscent JB extending into the mesotympanum on the PT-affected side. All initial surgeries were performed unilaterally as all subjects presented with unilateral PT.

**Table 1:** Summary of the 7 subjects who underwent revision operations or procedures for residual or recurrent pulsatile tinnitus after the initial surgery.

	Sex/age	Side	1st surgery	Causes	Materials	Residual/recurrent PT (duration)	2nd surgery	Causes	Materials	Follow-up duration after the revision surgery
1	F/40	R	SS resurfacing	SS-div	Tachocomb, T-fascia, Bone cement	Recurrent (8mo)	SS resurfacing	Recurrent diverticulum	Tachosil, T-fascia, bone cement	2 yr. 3 mo
2	F/55	R	SS resurfacing	SS-div/SS-deh	T-fascia, Mastoid periosteum, Bone cement	Recurrent (3 yr)	SS reshaping	Recurrent diverticulum	Conchal cartilage, bone cement	4 yr. 4 mo
3	F/41	R	SS resurfacing	SS-div/SS-deh	Bone cement	Residual	SS reshaping	Incomplete surgical reconstruction	Cortical bone chip, T-fascia, bone cement	3 yr. 8 mo
4	F/51	R	JB resurfacing	HJBD	Bone dust, T-fascia, Conchal cartilage	Residual	JB resurfacing	Incomplete surgical reconstruction	Bone cement, T-fascia	9 yr
5	M/41	L	SS reshaping	SS-deh	bone chip, T-fascia, bone cement	Residual	Transarterial embolization	Concomitant ipsilesional dural AVF	–	9 yr
6	F/36	R	SS resurfacing	SS-div/SS-deh	Bone wax, fibrin glue	Recurrent (4 yr. 6mo)	SS reshaping	Recurrent diverticulum	Harvested cortical bone chip, bone cement, fibrin glue	9 yr
7	M/39	R	SS reshaping	SS-deh	Cortical bone chip, T-fascia, bone cement	Recurrent (5mo)	SS reshaping	Residual small diverticulum	Tachosil, T-fascia, bone cement	5 mo

SS, sigmoid sinus; JB, jugular bulb; div, diverticulum; deh, dehiscence; yr, year; mo, month; T-fascia, temporalis fascia; AVF, arteriovenous fistula.

### 3.2. Postoperative course

**Figure 2:**
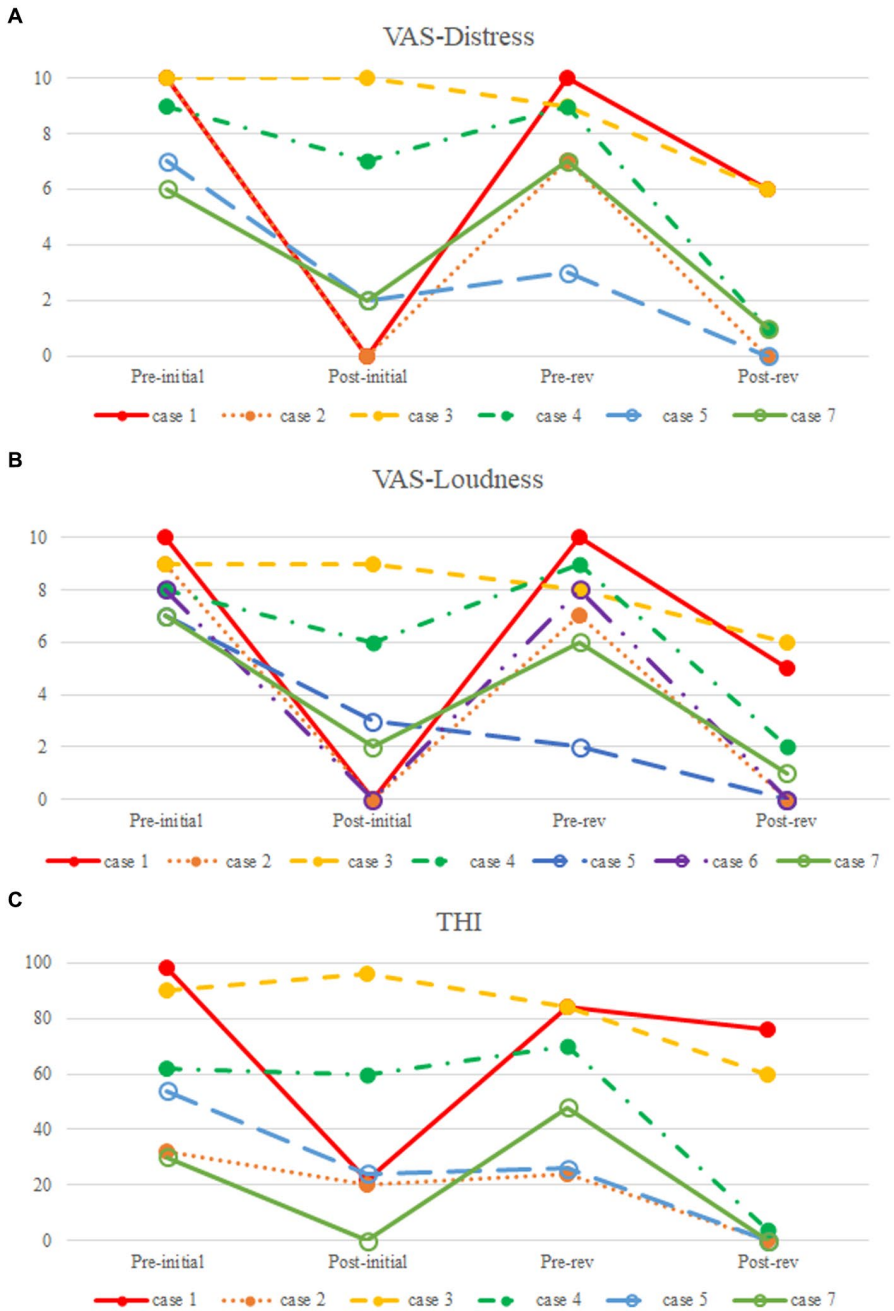
Comparison of pre-and postoperative subjective symptoms. **(A)** Visual analog scale (VAS)-scored tinnitus-related distress; **(B)** VAS-scored tinnitus loudness; **(C)** Tinnitus Handicap Inventory (THI).

**Figure 3:**
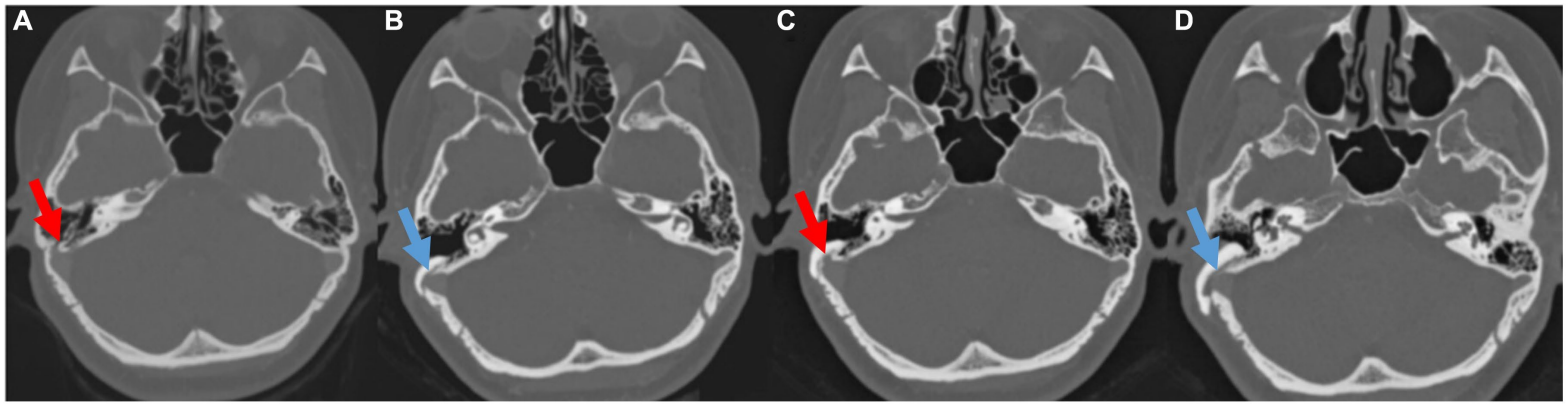
**(A,B)** Pre-and postoperative temporal bone computed tomography (TBCT) images of Subject 1 showing right sigmoid sinus diverticulum and successful resurfacing after the initial surgery. **(C)** Pre-revision surgery TBCT images of the same subject showing recurred diverticulum and **(D)** successfully resurfaced sigmoid sinus after the revision surgery.

**Figure 4:**
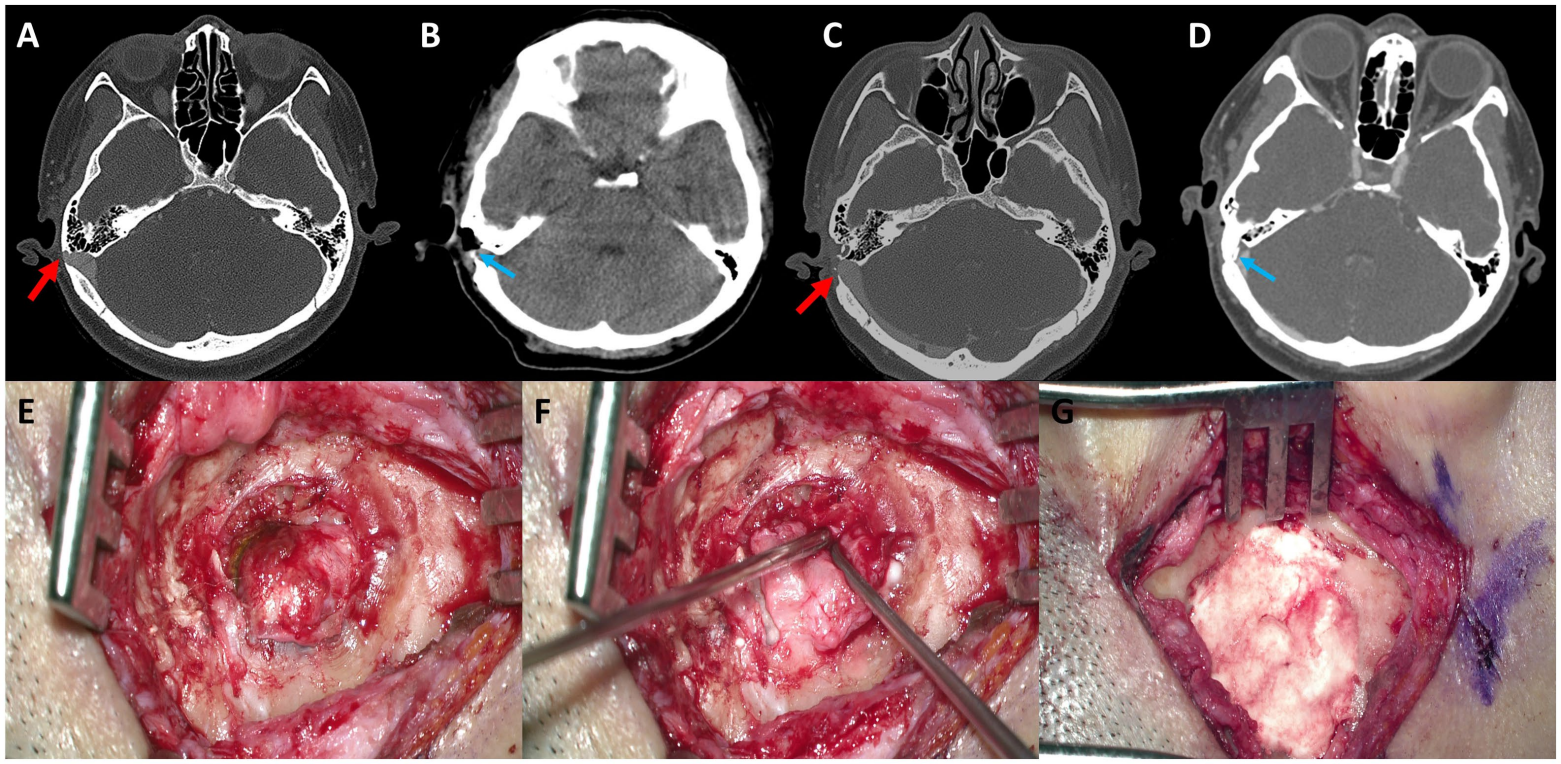
**(A)** Preoperative axial temporal bone computed tomography (TBCT) image of Subject 4 shows a huge diverticulum protruding through the mastoid air cells and cortical mastoid bone to the level of the subcutaneous soft tissue layer (arrow). **(B)** Postoperative axial TBCT image shows successful reduction of the diverticulum through transmastoid resurfacing with bone wax (arrow) and fibrin glue. **(C)** Follow-up TBCT axial image shows a huge diverticulum protruding through the mastoid air cells and cortical mastoid bone to the level of the subcutaneous soft tissue layer (arrow). **(D)** Again, the diverticulum is successfully reduced by transmastoid reshaping with harvested autologous cortical bone chips and bone cement (arrow). **(E)** The diverticulum was exposed to the mastoid cortex again. **(F,G)** Thus, revision reshaping of the sigmoid sinus diverticulum was performed with harvested autologous cortical bone chips and bone cement to reconstruct a secure sinus wall over the diverticulum.

**Figure 5:**
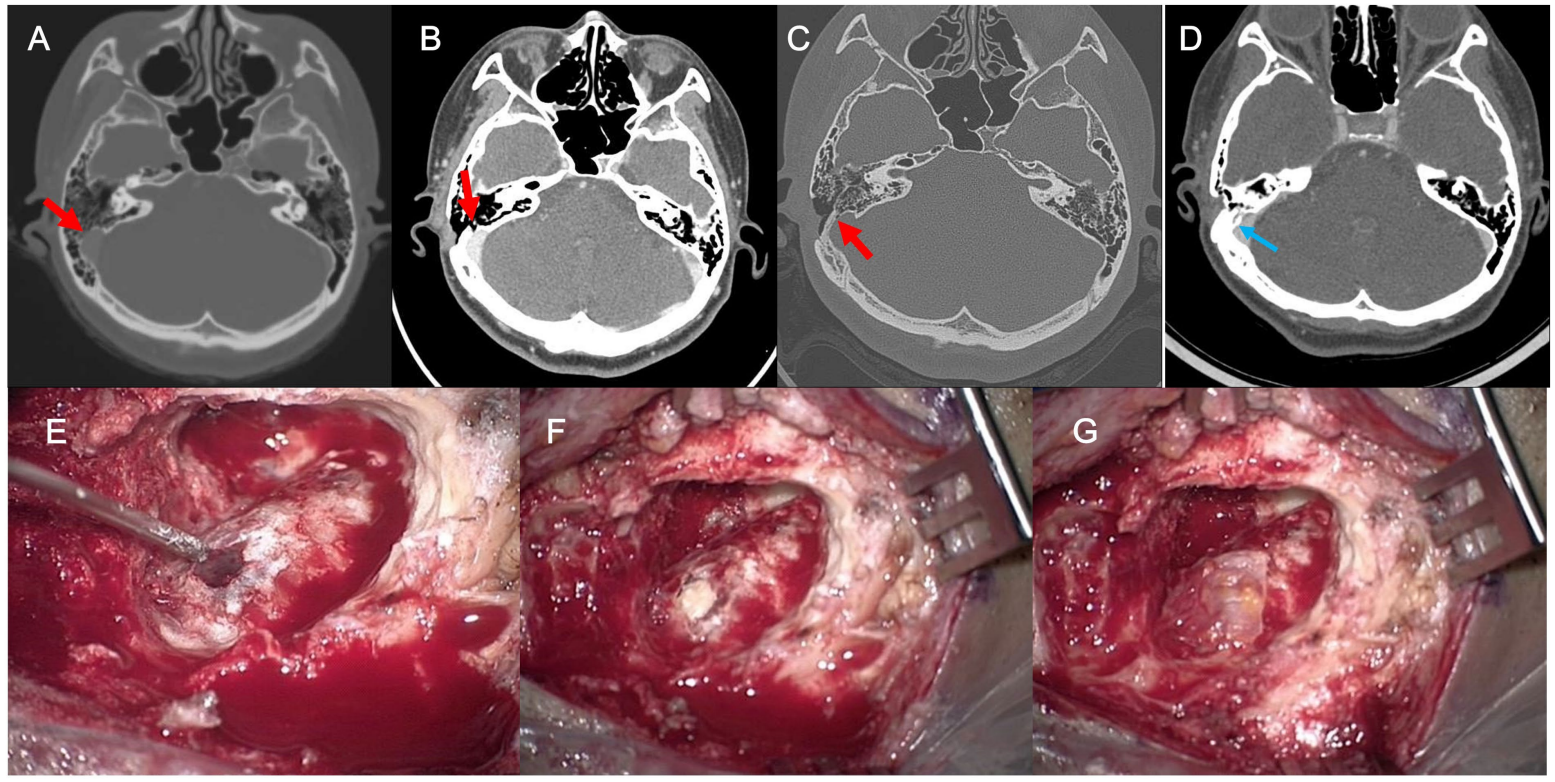
**(A)** Preoperative axial temporal bone computed tomography (TBCT) image of Subject 3 shows a diverticulum protruding through the mastoid air cells with bony dehiscence (arrow). **(B,C)** Follow-up postoperative TBCT axial image with and without contrast enhancement shows a residual bony dehiscence (arrow). **(D)** The dehiscence is successfully managed by transmastoid reshaping with a cortical bone chip, a piece of temporalis fascia, and bone cement (arrow). **(E)** The residual bony dehiscence was exposed. **(F,G)** Revision reshaping of the sigmoid sinus diverticulum with dehiscence was performed with a cortical bone chip, a piece of temporalis fascia, and bone cement to reconstruct a secure sinus wall.

**Figure 6:**
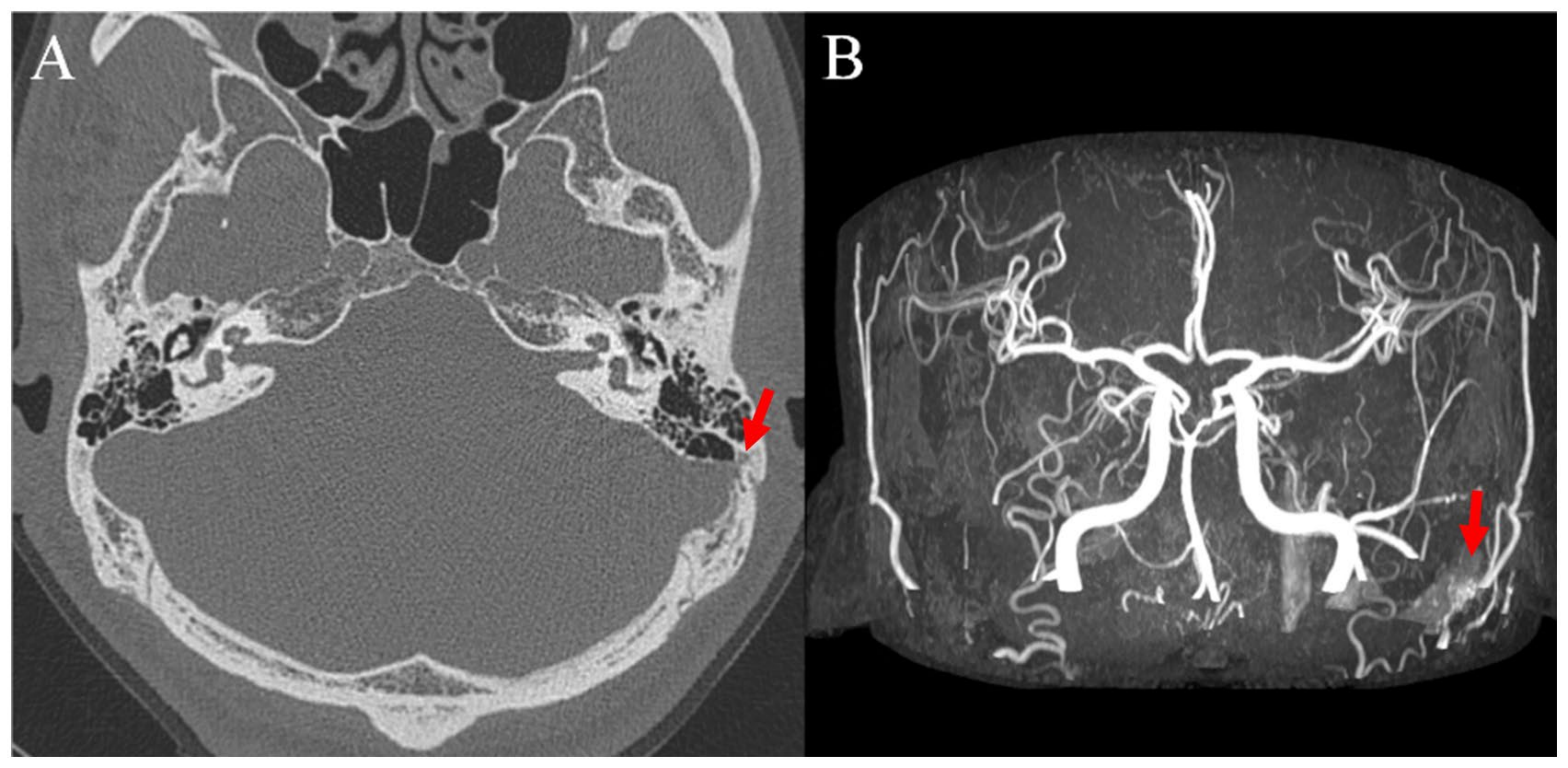
**(A)** Preoperative axial temporal bone computed tomography image showing left sigmoid sinus dehiscence and **(B)** postoperative brain magnetic resonance image with angiography image showing left dural arteriovenous fistula.

As illustrated in Figure 2, PT was much abated postoperatively in three of seven subjects (Subjects 1, 2, and 6), all of whom reported postoperative VAS loudness and distress scores of 0. One of seven subjects (Subject 6) did not complete the postoperative THI and VAS-scored tinnitus-related distress questionnaires. The median duration of PT recurrence in these three subjects was 36 months (range: 8–54 months). PT recurred in Subject 1 at 8 months after the initial resurfacing surgery of the SS-Div. Follow-up TB-CTA showed recurrent Div (Figure 3). Subject 2 complained of recurrent PT 3 years after initial treatment and presented with recurrent Div with bony Deh on follow-up TB-CTA. In Subject 6, PT returned to the preoperative level at 4.5 years postoperatively and TB-CTA indicated recurrent Div with bony Deh (Figure 4). Subject 3 showed no improvement of PT after initial SS resurfacing surgery at another hospital and TB-CTA showed residual SS-Deh (Figure 5). Subject 4 reported a slight improvement of PT after transcanal JB resurfacing using bone dust, temporalis muscle fascia, and conchal cartilage, but postoperative follow-up TB-CTA revealed residual Deh, probably due to limited exposure during transcanal microscopic resurfacing of the JB. Subject 5 reported partial resolution of PT even after meticulous reshaping of the SS-Deh and additional dAVF was found on MRA for residual PT (Figure 6). Subject 7 also reported only partial improvement of PT, followed by a return to the preoperative level after resurfacing of the SS-Deh, and postoperative follow-up TB-CTA revealed a residual small Div at the level of the transverse-SS junction.

### 3.3. Revision surgery/intervention

Subject 1 underwent SS resurfacing using Tachosil, a piece of temporalis muscle fascia, and bone cement, as in the initial surgery (Figure 3). Subjects 2, 3, 6, and 7 showed combined SS-Div/SS-Deh, and reshaping/resurfacing was performed in these four subjects using a cortical bone chip, a piece of temporalis muscle fascia, and bone cement (Figures 4, 5). Subject 4 underwent revision resurfacing of the remaining JB Deh using temporalis muscle fascia and bone cement. Subject 5 underwent additional transarterial embolization for the dAVF identified on MRA. PT resolved or improved markedly after revision surgery or intervention in five of seven subjects; the remaining subjects (Subjects 1 and 3) showed partial resolution of PT after revision operation (Figure 2). There were no immediate postoperative complications in this case series.

## 4. Discussion

This case series included subjects who underwent surgery for PT and then required revision surgery or intervention for residual or recurrent PT. Approximately 6% of subjects with PT who underwent surgery had remaining or recurrent symptoms. Three subjects showed complete resolution of PT after the first surgery but developed recurrent Div with or without Deh, three subjects had residual PT even after initial surgical reconstruction, and one subject had concomitant unidentified ipsilesional dAVF after undergoing surgical treatment for SS-Deh. The PT in these cases mostly improved with the application of appropriate surgical methods, reconstruction with more durable materials, and resolution of multiple etiologies.

Unlike non-pulsatile tinnitus, which is mostly associated with hearing loss (18), PT can be caused by local turbulent blood flow resulting from changes in vascular hemodynamics, in turn resulting in disruption of laminar flow and serving as the source of PT (3, 19). Another mechanism is the transmission of blood flow vibration to the middle and inner ear through well-pneumatized mastoid air cells when there is Deh in the vascular wall (15). Consequently, to alleviate PT, it is essential to address the vascular irregularities responsible for inducing local turbulence in the blood flow, with the goal of restoring laminar flow. In addition, reinforcing the bony walls serves to prevent sound transmission.

In cases with an intact but remodeled vascular wall, adequate soundproofing can be achieved through external reduction and resurfacing of the Div. However, in instances of bony wall Deh, resurfacing alone may not be sufficient. The underlying mechanisms for vascular wall Deh are not yet clearly understood, but there is a possibility that turbulence caused by rapid flow in the dominant venous system, as per Poiseuille’s law, may lead to erosion of the bony wall (3, 7). Therefore, in cases where only resurfacing is performed, there is a possibility of symptom recurrence due to continuous venous pressure on the surgically reconstructed area. In cases of bony wall Deh, reshaping through compression using materials such as bone chips or cartilage can decrease venous blood flow, thus effectively minimizing turbulence in blood flow and reducing the likelihood of recurrence (12). Subjects 2, 3, and 6 in our study initially underwent resurfacing for SS-Div with SS-Deh, but reshaping was performed later due to persistent or recurrent symptoms.

To avoid displacement or breakage of the materials used for surgical reconstruction of SS-Div or SS-Deh, it is imperative to use durable materials. The use of materials such as bone dust or bone pâté for resurfacing may not suffice for solid reconstruction, and it is recommended that materials with good durability be used, such as bone cement (7, 10, 12, 15). The use of bone cement has been reported to provide a higher success rate compared to other materials (3, 6). Subjects 4 and 6 in this case series initially underwent surgical reconstruction using materials such as bone dust and bone wax, resulting in incomplete reconstruction and residual PT in Subject 4, and subsequent relapse in Subject 6. Revision surgeries using bone cement ensured secure reconstruction and symptom resolution in both cases.

PT can have various vascular etiologies. Arterial abnormalities that may contribute to PT include carotid artery stenosis, vertebral or carotid dissection, fibromuscular dysplasia, aneurysm, aberrant internal carotid artery, and dAVF (20). Venous abnormalities that can cause PT include dural sinus stenosis, SS wall abnormalities, jugular vein anomalies, and emissary vein anomalies (20). These abnormalities can lead to changes in blood flow and pressure that result in PT. It should be noted that the coexistence of multiple underlying pathologies may also contribute to the development of this condition. Subject 5 was initially diagnosed with a SS-Deh as the causative lesion of PT on TB-CTA, and an appropriate reshaping procedure was performed using bone cement. Despite some improvement, PT persisted. Further evaluation by MRA revealed the presence of dAVF on the ipsilateral side, and the remaining symptoms disappeared following transarterial embolization. Li et al. (10) also reported cases where symptoms persisted even after correcting SS anomalies caused by accompanying JB anomalies and venous sinus stenosis. Therefore, it is imperative to conduct meticulous and comprehensive evaluations for possible multiple etiologies prior to surgical intervention.

Subject 1 was diagnosed with SS-Div and underwent a resurfacing procedure using bone cement, resulting in postoperative symptom resolution. However, subsequent to a rapid increase in weight, the subject reported recurrent PT, which was confirmed by TB-CTA imaging to be caused by recurrence of the Div. Previous reports have documented a correlation between idiopathic intracranial hypertension (IIH) and SS-Div and SS-Deh (11, 21, 22). IIH is also considered one of the underlying causes of PT (23, 24). IIH is more prevalent among females, and weight gain is a well-established risk factor (25). In addition, elevated body mass index and female predominance have also been observed in patients with SS-Div or SS-Deh, suggesting a relationship between the two conditions (26). In regard to Subject 1 in our case series, it may be hypothesized that weight gain may have played a role in the occurrence of IIH, which in turn contributed to the relapse of SS-Div. In addition, after the initial surgery, the VAS-scored loudness and distress decreased from 10 to 0, and the THI score decreased from 98 to 22. However, after revision surgery, the VAS-scored loudness and distress decreased from 10 to 6 and 5, respectively, and the THI score decreased from 92 to 74, reflecting some improvement but indicating residual subjective discomfort. The residual symptoms after revision surgery may have been attributable to the underlying IIH.

While research on revision surgery for PT remains limited, a study conducted by Li et al. (10) examined the causes of revision in seven cases following reconstruction of the sigmoid sinus wall in patients with PT. Among the seven cases, five were early cases with limited surgical experience, and they showed symptom improvement after undergoing revision surgery primarily due to incomplete elimination of sigmoid sinus wall dehiscence and sigmoid sinus diverticulum. The remaining two cases exhibited symptom improvement after treatment for abnormalities such as abnormal diploic veins and venous sinus stenosis, in addition to sigmoid sinus reconstruction. Similarly, in our own cases, there were instances where revision surgery was necessary due to residual dehiscence resulting from incomplete surgical reconstruction. Additionally, we encountered a case requiring transarterial embolization for a dAVF following SS reshaping. These findings highlight the importance of skilled surgeons thoroughly exposing the lesions and employing appropriate surgical methods to achieve complete reconstruction. Furthermore, when investigating the underlying causes of PT, it is crucial to always consider the possibility of multiple etiologies. Furthermore, in our cases, PT recurrence occurred when using materials prone to displacement or breakage, such as bone dust or bone pate. Therefore, utilizing durable materials for reconstruction becomes pivotal in enhancing the success rate of surgical interventions.

This case series highlighted the importance of considering multiple etiologies, conducting a thorough evaluation, using durable materials for the surgical management of PT, and applying an appropriate surgical method. It also suggested the need to consider risk factors, such as weight gain, in the management of SS-Div and SS-Deh in association with IIH. In the present study, about 6% of subjects experienced recurrence or residual symptoms after initial surgical treatment necessitating revision operation or additional procedures. The observations in our case series showed that even if recurrence occurs or symptoms persist, proper management using appropriate surgical techniques and materials during revision surgery can effectively resolve the issue, taking the possibility of coexisting pathologies into account. Further research is needed to understand the underlying mechanisms of bony wall Deh, and to develop effective measures to prevent recurrence.

The present case series is limited in terms of its sample size, although 101 operations for SS-Deh or SS-Div are one of the largest case series to the best of our knowledge. The limited sample size presents challenges in establishing conclusive findings regarding the overall population of patients with PT undergoing surgical interventions. In addition, the study’s ability to establish a causal relationship between weight gain and the relapse of sigmoid sinus diverticulum in subject 1 is limited, as the presence of other potentially contributing factors cannot be ruled out. Furthermore, since there are subjects who were followed up less than 1 year after the revisional surgery, a long-term follow-up is mandated to draw conclusions regarding the surgical success. To validate the findings of this case series, it will be necessary to conduct further studies with larger sample sizes and longer follow-up duration.

## 5. Conclusion

The possibility of recurrence should be taken into account when performing surgical intervention in patients with PT. The likelihood of recurrence can be minimized through a comprehensive evaluation to identify possible multiple etiologies, and by using durable materials and appropriate surgical methods.

## Data availability statement

The original contributions presented in the study are included in the article/supplementary material, further inquiries can be directed to the corresponding author.

## Ethics statement

The studies involving human participants were reviewed and approved by Institutional Review Board of the Clinical Research Institute at Seoul National University Bundang Hospital. Written informed consent for participation was not required for this study in accordance with the national legislation and the institutional requirements.

## Author contributions

YS, HL, and J-JS led the analysis and interpretation of the result and drafted the first manuscript. HL and J-JS conceived the investigation, and revised the manuscript for important intellectual content. YS, HL, S-MP, DK, J-WK, and J-JS contributed to all aspects of the investigation, including methodological design, data collection and analysis, interpretation of the results, and revision of the manuscript for important intellectual content. All authors contributed to the article and approved the submitted version.

## Funding

This work was supported by grants from the National Research Foundation of Korea (NRF) grant funded by the Korean government (MSIP) (grant No. NRF-2022R1A2B5B02002139 to J-JS), from the Korea Health Technology R&D Project through the Korea Health Industry Development Institute (KHIDI) funded by the Ministry of Health and Welfare (grant No. HI21C1574 to J-JS), and from Seoul National University Bundang Hospital (grant No. 14-2022-0038 to J-JS).

## Conflict of interest

The authors declare that the research was conducted in the absence of any commercial or financial relationships that could be construed as a potential conflict of interest.

## Publisher’s note

All claims expressed in this article are solely those of the authors and do not necessarily represent those of their affiliated organizations, or those of the publisher, the editors and the reviewers. Any product that may be evaluated in this article, or claim that may be made by its manufacturer, is not guaranteed or endorsed by the publisher.
